# Pharmaceutical stability of colloidal saccharated iron oxide injection in normal saline

**DOI:** 10.1186/s40780-018-0116-0

**Published:** 2018-07-25

**Authors:** Daiki Hira, Asami Suzuki, Yusuke Kono, Kosuke Shimokawa, Serika Matsuoka, Ken-yuh Hasumoto, Hiroyuki Kawahara, Masahide Onoue, Takuya Fujita, Tomonobu Okano, Mikio Kakumoto

**Affiliations:** 1 Laboratory of Clinical Pharmacy, College of Pharmaceutical Sciences, Ritsumeikan University, 1-1-1 Noji-higashi, Kusatsu, Shiga 525-8577 Japan; 2 Department of Pharmacy, Shiga University of Medical Science Hospital, Seta Tsukinowa-Cho, Otsu, Shiga 520-2192 Japan; 3 Laboratory of Molecular Pharmacokinetics, College of Pharmaceutical Sciences, Ritsumeikan University, 1-1-1 Noji-higashi, Kusatsu, Shiga 525-8577 Japan; 4 Department of Pharmacy, Kitano Hospital The Tazuke Kofukai Medical Research Institute, 2-4-20 Ohgimachi, Kita-ku, Osaka, 530-8480 Japan

**Keywords:** Incompatibility, Colloid, Iron injection, Pharmaceutical stability, Electrolyte, Diluent

## Abstract

**Background:**

Colloidal saccharated iron oxide injection is used for the treatment of iron deficiency anemia in patients with a poor oral intake. Because of the poor stability of the colloid particle, there have been concerns regarding its compatibility with various injections in clinical practice. To assess the stability of colloidal saccharated iron oxide in normal saline as a diluent, pharmaceutical stability analyses were conducted using various concentrations of glucose and sodium chloride (NaCl).

**Methods:**

Colloidal saccharated iron oxide injection was diluted in three different diluents (5% glucose solution, normal saline, and 10% NaCl solution), and its appearance, colloid particle diameter, and pH were assessed. Free iron ions, which cause adverse effects, such as nausea and vomiting, were separated from the colloid particle using a dialysis membrane for 24 h, and their concentration was determined.

**Results:**

No difference in the appearance, colloid diameter, and free iron ion fraction was observed after dilution in 5% glucose solution and normal saline. Conversely, an increased colloid aggregation and iron ion release were observed after dilution in 10% NaCl solution. Although iron colloid is unstable in acidic conditions (pH 4.0–6.0), normal diluents such as 5% glucose and normal saline did not cause colloid destabilization by pH change (pH > 8.0).

**Conclusion:**

Normal saline may be used as a diluent of colloidal saccharated iron oxide injection as well as glucose solution, which is recommended by the pharmaceutical company. Therefore, normal saline can be used as a diluent of colloidal saccharated iron oxide injection in patients with an underlying disease, such as diabetes mellitus, who are difficult to use glucose solution as a diluent.

## Background

Colloidal saccharated iron oxide injection is used for the treatment of iron deficiency anemia in patients with a poor oral intake [1–5]. Because of the colloid’s stability, there have been concerns regarding its compatibility with various injections in clinical practice [6–8]. The disruption of the colloidal iron particle enhances the isolation of iron ions, resulting in adverse effects such as nausea and vomiting [9–11]. Therefore, the pharmaceutical company recommends the colloidal saccharated iron oxide (Fesin®) to be diluted in glucose solution. Conversely, in clinical practice, using glucose solution as a diluent is difficult in patients with an underlying disease such as diabetes mellitus because additional glucose injection may cause a poor control of their disease. In such patients, normal saline [0.9% sodium chloride (NaCl)] has often been used as a diluent of colloidal saccharated iron oxide. Although electrolytes are well-known destabilizers of colloidal particles, normal saline is frequently used as a representative isotonic diluent. However, information regarding the pharmaceutical stability of colloidal saccharated iron oxide in various diluents, including normal saline but excluding glucose solution, has been lacking. In this study, the stability of colloidal saccharated iron oxide in normal saline as a diluent was assessed by conducting pharmaceutical stability analyses using various concentrations of glucose and NaCl.

## Methods

Fesin® (colloidal saccharated iron oxide for injection, 40 mg/2 mL; Nichi-Iko Pharmaceutical Co., Ltd., Toyama, Japan), glucose injection (5, 10, and 20%; Otsuka Pharmaceutical Factory, Inc., Tokushima, Japan), and normal saline injection (0.9% NaCl; Otsuka Pharmaceutical Factory, Inc., Tokushima, Japan) were purchased from the respective pharmaceutical companies. The other reagents and solvents, such as NaCl, 1,10-phenanthroline, sulfuric acid, and hydroxylamine chloride were of analytical grade.

### Influence of diluents on appearance and particle diameter

The appearance of Fesin® 1 A (40 mg/2 mL) in 50 mL of three different diluents (5% glucose solution, normal saline, and 1.25–10% NaCl solution) was observed at 0 and 24 h after dilution. Although the pharmaceutical company recommends the use of a 10–20% glucose solution as a diluent in the Fesin® package, 5% isotonic glucose solution is the standard diluent used for Fesin® in clinical practice. Therefore, a 5% glucose solution was used as a control diluent. The diluted samples were stored at room temperature until assessment. Dilution conditions were set at frequently usage in clinical setting. The influence of pH change on its appearance was assessed between pH 4.0 and 10.0, adjusted by the addition of 0.1 M HCl after dilution with distilled water. To assess the participation of colloid particles, all samples were centrifuged for 2 min at 3500 rpm (1100×g) before the assessment.

In addition to the evaluation of appearance, colloid particle diameters were determined at 0–24 h after dilution using various diluents (5% glucose, normal saline, 1.25–10% NaCl, and pH 4.0–10.0 solutions) using dynamic light scattering analysis (Zetasizer nano ZS; Malvern Instruments, Ltd., Worcestershire, UK) at 25 °C.

### Influence of diluents on pH change

pH changes were measured at 0–24 h after diluting Fesin® 1A in 50 mL of three diluents (5% glucose solution, normal saline, and 10% NaCl solution). The diluted samples were stored at room temperature until measurement. The influence on pH was also assessed at various solute concentrations (glucose, 0–20%; NaCl, 0–10%) and volumes (10–50 mL) of diluents at 0 and 24 h after dilution.

### Free iron ion released from iron oxide colloid particles

Free iron ions, which cause adverse effects such as nausea and vomiting [9–13], were separated from colloid particles (approximately 10 nm) using dialysis membrane (Spectra Por® 3.5 kDa; pore size, < 2 nm; Spectrum, Inc., OH, USA). One ampule of Fesin® was diluted in 50 mL of three diluents (5% glucose solution, normal saline, and 10% NaCl solution) and used as the testing sample. Iron ions are adsorbed on the surface of the cellulose dialysis tube [14, 15]; hence, to saturate the tube surface by iron ion adsorption, a certain concentration of the standard iron ion solution (2 μg/mL) was added to both the inside (2 mL testing sample solution) and outside (10 mL blank solution) of the dialysis tube (70 mm × 11.5 mm i.d.). After stirring for 24 h at room temperature, the amount of free iron ions in the outside solution was determined using the colorimetric o-phenanthroline method [16]. Free iron ions derived from colloidal saccharated iron oxide were calculated from the difference in the outside iron ion concentrations between the sample and blank solutions. The calibration curve ranged from 0.16 to 10 μg/mL. The colorimetric o-phenanthroline method is briefly summarized as follows: 25% sulfuric acid, 10 μL; 10% hydroxylamine chloride, 20 μL; acetate buffer, 40 μL; and 0.5% 1,10-phenanthroline, 40 μL were added and mixed to 1 mL of sample solution collected from the outside of the dialysis tube [17]. After 15 min of incubation in dark at room temperature, the resultant compound is determined at 510 nm.

### Statistical analysis

Differences in each parameter among the three diluents were assessed using one-way analysis of variance (ANOVA) and Tukey’s multiple comparisons (*P* < 0.05). All statistical analyses were performed using EZR (Saitama Medical Center, Jichi Medical University), which is a graphical user interface for R (the R Foundation for Statistical Computing, version 3.3.2) [18].

## Results

### Influence of diluents on particle appearance and diameter

**Fig. 1:**
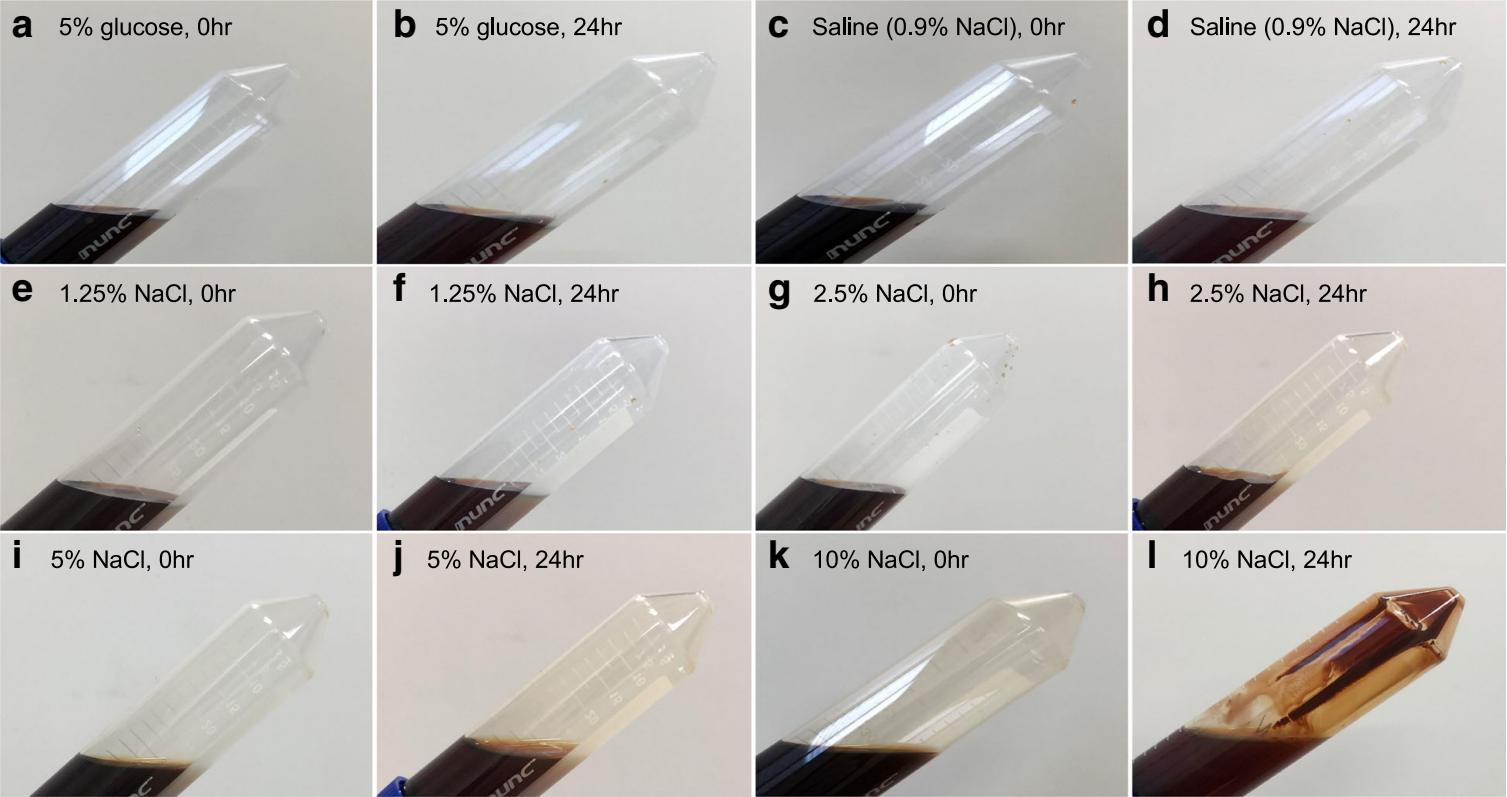
Time-dependent appearance change of colloidal saccharated iron oxide in three different diluents. 5% glucose solution (**a**, **b**), normal saline (0.9% NaCl solution) (**c**, **d**), 1.25% (**e**, **f**), 2.5% (**g**, **h**), 5% (**i**, **j**), and 10% (**k**, **l**) NaCl solution at 0 (**a**, **c**, **e**, **g**, **i**, **k**) and 24 (**b**, **d**, **f**, **h**, **j**, **l**, **h**)

**Fig. 2:**
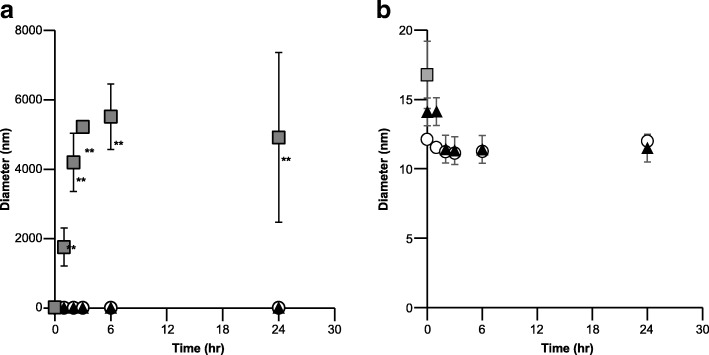
Colloid particle diameter after dilution by various diluents. **b** An enlarged view of (**a**) gray squares, 10% NaCl solution; black triangles, normal saline (0.9% NaCl solution); and white circles, 5% glucose solution. **P < 0.01 vs. 5% glucose and normal saline (ANOVA with Tukey’s test)

Figure 1 shows the change of appearance at 0 and 24 h after dilution in three diluents (5% glucose, normal saline, and 10% NaCl). There was no obvious difference in 5% glucose and normal saline at both time points. Conversely, dilution in 2.5 and 5% NaCl caused a slight precipitation at 24 h; furthermore, dilution in 10% NaCl resulted in a suspension at 0 h and a drastic precipitation at 24 h. The similar results were observed in particle diameter (Fig. 2). Significantly larger particles were detected in dilution with 10% NaCl, whereas colloid diameters in 5% glucose and normal saline were 10–15 nm at 24 h after dilution.

### Influence of diluents on pH change

**Fig. 3:**
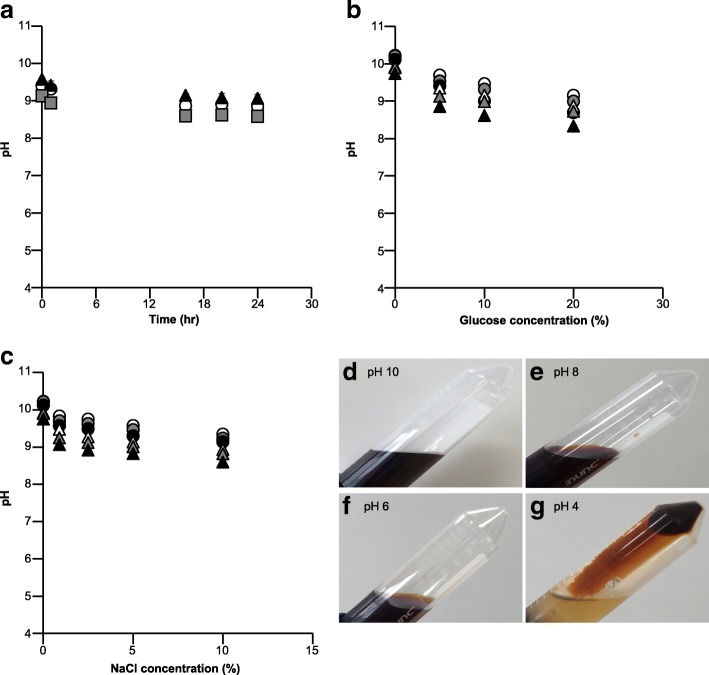
**a** Time-dependent pH change in various diluents. Gray squares, 10% NaCl; black triangles, normal saline; and white circles, 5% glucose. **b**, **c** Relationship between pH and solute concentration in different diluents. Volumes of diluents are represented by different colors. White, gray, and black plots indicate 10 mL, 20 mL, and 50 mL, respectively. Circles and triangles indicate 0 and 24 h after dilution, respectively. **d**–**g** Colloid stability in different pH conditions

**Fig. 4:**
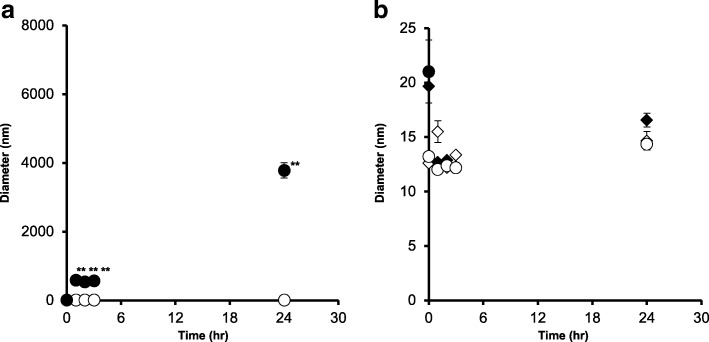
Colloid particle diameters after dilution at different pH levels. **b** An enlarged view of (**a**) black circles, pH 4.0; white circles, pH 6.0; black triangles, pH 8.0; and white triangles, pH 10.0. **P < 0.01 vs. pH 6.0–10.0 (ANOVA with Tukey’s test)

Figure 3a shows pH change after dilution. There were no time-dependent trends of pH change in all diluents, while pH in all diluents decreased from pH 9.0–9.7 to pH 8.5–9.3 after dilution. The influence of solute concentration and volume of diluents were also investigated (Fig. 3bc). A higher solute concentration and larger volume of diluent resulted in lower pH; however, the lowest pH in the testing conditions was pH 8.0. Regarding the colloid appearance, iron colloid was unstable in acidic conditions (pH 4.0–6.0; Fig. 3d–g). Figure 4 reports colloid particle median diameters at different pH levels and appearance change. Significantly larger particles were detected at pH 4.0, whereas colloid particle diameters at pH 6.0–10.0 were between 10 and 20 nm at 24 h after dilution.

### Free iron ions released from iron oxide colloid particles

**Fig. 5:**
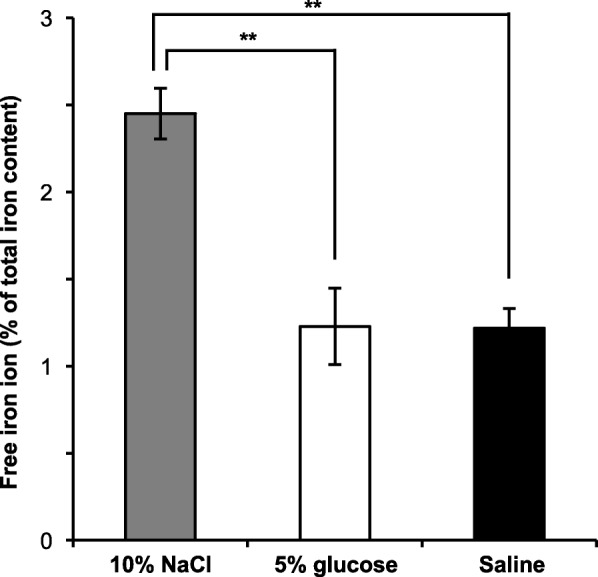
Amount of free iron ions released from iron colloid particles at 24 h after dilution. Data are presented as mean ± SD (*n* = 3). **P < 0.01 vs. 5% glucose and normal saline (ANOVA with Tukey’s test)

Figure 5 demonstrates the amount of free iron ions released from iron oxide colloid particles in the three tested diluents. 10% NaCl produced a two-fold greater free iron ion isolation ratio than 5% glucose and normal saline. Conversely, there was no difference between 5% glucose and normal saline.

## Discussion

In this study, we investigated the pharmaceutical stability of a colloidal saccharated iron oxide injection in normal saline. The results of appearance, particle diameter, and free iron ion detection tests suggested no significant difference in the pharmaceutical stability between 5% glucose and normal saline, except for 10% NaCl. The destabilization of iron colloid caused by the higher amount of electrolyte derived from 10% NaCl leads to the aggregation and enlargement of colloidal particles and in a higher ratio of free iron ion release. From a therapeutic viewpoint, pH and electrolyte levels at the site of colloid administration are important concerns, because of their effects on the surface potential of the colloid particles, which determines the colloid stability [6]. In the Fesin® package insert information, the pharmaceutical company also calls the attention of medical professionals to these drug interactions. Herein, pH test revealed that the colloid appearance and diameter were stable between pH 8.0–10.0; however, they were unstable under acidic conditions (pH 4.0–6.0). Therefore, in the clinical setting, normal diluents such as 5% glucose and normal saline do not cause colloid destabilization by pH change (pH > 8.0 in clinical condition). Conversely, electrolytes are well-known destabilizers of colloidal particles [6, 19, 20]. NaCl is a typical electrolyte, which may cause colloid destabilization; however, low NaCl concentration, such as in normal saline (0.9%), may not cause the destabilization. Hence, a high NaCl concentration (2.5–10%) is necessary to decrease the stability of colloidal iron injection. From the viewpoint of clinical usage, there is no pharmaceutical destabilization of colloidal saccharated iron oxide at low NaCl concentrations (such as normal saline, 0.9%). Normal saline should be a suitable diluent for colloidal saccharated iron oxide in patients with diabetes mellitus. Thermal conditions are critical in evaluating colloid stability. Shah et al. reported that there was no change in the molecular weight of iron colloid after heating at 30 °C and 40 °C for 35 days. However, the iron colloid destabilized when heated at 50 °C and 70 °C post 7 days [6]. Based on these information, room temperature is more severe condition than cool condition (4 °C). Therefore, in our study, the assessment of stability at room temperature can be considered robust according to common clinical conditions. A limitation of our study is that no clinical data were available to assess the safety and efficacy of normal saline as a diluent of colloidal saccharated iron oxide. Further clinical study should be conducted to address this concern.

## Conclusion

This study suggests that normal saline can be used as diluents of colloidal saccharated iron oxide as well as glucose solution, which is recommended by the pharmaceutical company. In clinical practice, glucose solution is difficult for use as a diluent in patients with an underlying disease such as diabetes mellitus. In such patients, normal saline is advantageous to decrease the risk of poor diabetes mellitus control. Although further clinical study should be conducted to demonstrate its safety and efficacy, this is the first useful information about the diluent selection for colloidal saccharated iron oxide injection.
